# Bridging the educational gap in terms of digital competences between healthcare institutions’ demands and professionals’ needs

**DOI:** 10.1186/s12912-023-01284-y

**Published:** 2023-04-27

**Authors:** O. Navarro-Martínez, J. Igual-García, V. Traver-Salcedo

**Affiliations:** 1 Department of Nursing, Catholic University of Valencia, Valencia, Spain; 2 Instituto de Telecomunicaciones y Aplicaciones Multimedia (ITEAM), Departamento de Comunicaciones, Universitat Politècnica de València, Valencia, Spain; 3 Instituto ITACA, Universitat Politècnica de València, Calle Espartero 7, Valencia, Spain

**Keywords:** Ehealth, Health professions, Computer literacy, Organizational culture, Knowledge management, Nursing, Leadership

## Abstract

**Background:**

Healthcare professionals with insufficient digital competence can be detrimental to patient safety and increase the incidence of errors. In order to guarantee proper care, healthcare organizations should provide opportunities to learn how to use technology, especially for those professionals who have not received training about this topic during their undergraduate studies.

**Objective:**

This exploratory study aimed to conduct surveys among Spanish healthcare professionals to determine whether their organisations had trained them in the use of healthcare technology and the areas where most emphasis was placed.

**Methods:**

1624 Spanish healthcare professionals responded to an ad hoc online survey 7 questions related to the digital skill training offered by the healthcare organisations they work for.

**Results:**

Nurses were the most widely represented group, making up 58.29% of the total, followed by physicians namely 26.49%. Only 20% of the nurses surveyed had received some training from their institution related to healthcare technology. According to the participants’ responses, physicians received significantly more training in this area than nurses. Training related to database searching for research purposes or computer management followed the same trend. Nurses also received less training than physicians in this area. 32% of physicians and nurses paid for their own training if they did not receive any training from institutions.

**Conclusions:**

Nurses receive less training, on topics such as database searching or management, from the healthcare centres and hospitals where they work. Moreover, they also have fewer research and digital skills. Both of these factors may lead to deficits in their care activities, and have adverse effects on patients. Not to mention fewer opportunities for professional progress.

## Introduction

In order to reach patients, save costs and streamline procedures, various public and private organisations advocate developing and implementing digital health systems, or *ehealth*, in hospitals and healthcare centres among healthcare professionals [1–3].

However, few healthcare systems have committed to educating, training and updating their healthcare professionals as for these digital competences. Similarly, very few healthcare professionals can apply such competences, even when given institutional support [4].

The Committee on Digital Skills for Healthcare Professionals concluded that more than 80% of healthcare professionals had insufficient or inadequate training in ehealth or mhealth (digital health mediated by mobile technology) [5]. Equivalently, WHO Atlas of National eHealth profiles [6] placed Spain in a medium-low level in terms of eHealth capacity building for healthcare professionals.

Does this mean that these professionals lack sufficient digital skills in order to recommend these resources to patients or, that, they do not receive enough institutional support from their organisations to train and use them in their professional lives? When it comes to nurses, for example, the degree syllabus that they follow in Spain does not include subjects that cover all the required areas to be digitally “competent”, as recommended by various international organisations and related publications [7–10]. It is important to remember that insufficient digital competence by healthcare professionals can be detrimental to patient safety and increase the incidence of errors [4]. In fact, some studies have reported errors of up to 35% related to digital medical prescriptions due to unfamiliarity with the software or a lack of digital skills [11].

In addition, there is evidence that nurses’ technological skills influence the frequency of their technology use, i.e., the better their skills, the more they are used [4]. However, other research shows that the motivation to learn and convey is not always directly related to the training received, but it is also influenced by other factors such as their work climate and institutional support [12]. Consolidating their learning requires opportunities to apply what has been learned in the professional environment [13] and this is where healthcare institutions play an important role [14]. Therefore, healthcare organisations are responsible for providing sufficient resources, equipment, and space for the use of technology, as well as providing healthcare professionals with the time and opportunities to learn how to use them [4], especially for those nurses who have not received training in this area during their undergraduate studies [15].

The aim of this exploratory study was, thus, to conduct a survey among Spanish healthcare professionals to find out whether their healthcare organisations (hospitals, health centres, and other services) train them in the use of healthcare technology and to identify the areas where most emphasis is placed. We were also interested in identifying any particular differences in terms of training between professional categories or areas.

Our preliminary assumption was that few healthcare professionals currently receive training about digital skills from their organisations in Spain.

## Materials and methods

An online survey was launched, all types of healthcare professionals working in Spain could respond, in order to obtain information on the digital skills training they had received from their healthcare organisations and institutions. Responses were accepted from physicians, nurses, midwives, physiotherapists, auxiliary nursing care technicians (TCAE), pharmacists, psychologists, health emergency technicians (TES) and others.

The ad hoc questionnaire was developed, revised and agreed upon by an expert panel composed of three researchers. These three experts belong to multidisciplinary fields; health, technology and engineering, and helped to define the questions asked as well as making them more understandable.

This questionnaire is based on the conclusions of Konttila[4] and Kaihlanen [15]: it is healthcare organisations’ duty to ensure the digital literacy of their professionals.

The survey only included seven questions from two areas: professional data and training received. In the former, the participants could add categories other than those offered on the list. All questions were compulsory and therefore no questions were left unanswered. The estimated time taken when filling out the questionnaire was 4 min. The complete survey can be seen in Table 1. The inclusion criteria for participation in the survey was to be a healthcare professional who is currently working. Therefore, responses from retired or unemployed professionals, students or administrative staff were excluded.

As an introduction before the survey, the text regarding the study purpose appears, where a reference about the approval of the ethics committee, and how data from the survey was going to be handled is also made. After acknowledging and accepting that, the healthcare professional was able to carry out the survey.

We used the following formula to calculate the necessary sample size to estimate a population proportion (p) of a large population with 95% confidence and a margin of error no larger than e=+/-5% for the most uncertain case (the worst-case scenario) p = 50%: N = z^2*p*(1-p)/e^2 [16]. Based on this formula choice, 384 responses were needed.

**Table 1:** Completed survey

**Professional data**
● Professional profile
o Physician
o Nurse
o Midwife
o Physiotherapist
o TCAE
o Pharmacist
o Psychologist
o Dentist
o TES
o Other
● You are currently working in:
o Primary Care
o Hospital
o Residence
o University
o Other
**Training received**
● Question 1: Have you recently received any training related to the use of technology in the healthcare field from your company or organisation?
● Question 2: Have you recently received any training related to the creation of healthcare content for social networks, videos, and videoconferences?
● Question 3: Have you recently received any training related to database searches from your company or organisation?
● Question 4: Have you received any training related to computer management from your company or organisation?
● Question 5: Have you paid for any of the courses mentioned above?

This questionnaire was developed on Google Forms, which stores the responses given and facilitates their analysis. The participants clicked on a link for access and were taken directly to the survey. Registration or personal details were not required.

In order to reach different types of healthcare professionals, the survey was sent via Instagram, Facebook, Twitter, and LinkedIn. The aim was to reach different profiles in terms of age, gender, digital skills, profession, etc. The only requirement was to be a working healthcare professional. The survey was active online from 14th July to 19th October 2021. Over these three months, frequent reminders were sent through the social networks mentioned above.

### Ethical approval

to conduct this survey was obtained from the Research Ethics Committee of the Polytechnic University of Valencia, Spain (P4_25_07_18). No personal data was collected. Participation was free of charge and completely voluntary.

Different tests were applied to check whether the results were statistically significant (P < 0.05). Most variables were dichotomous. We used the chi-square as the test statistic with the corresponding degrees of freedom, depending on the dimensions of the contingency table.

## Results

A digital media survey was conducted to assess whether health institutions currently offer nurses and other health professionals training as well as what content is provided. The questions on training received were answered Yes or No, although the option “Other” was included.

During this period, 1624 responses were received, and 80 of them were eliminated because they did not meet the necessary criteria, mentioned above.

**Fig. 1:**
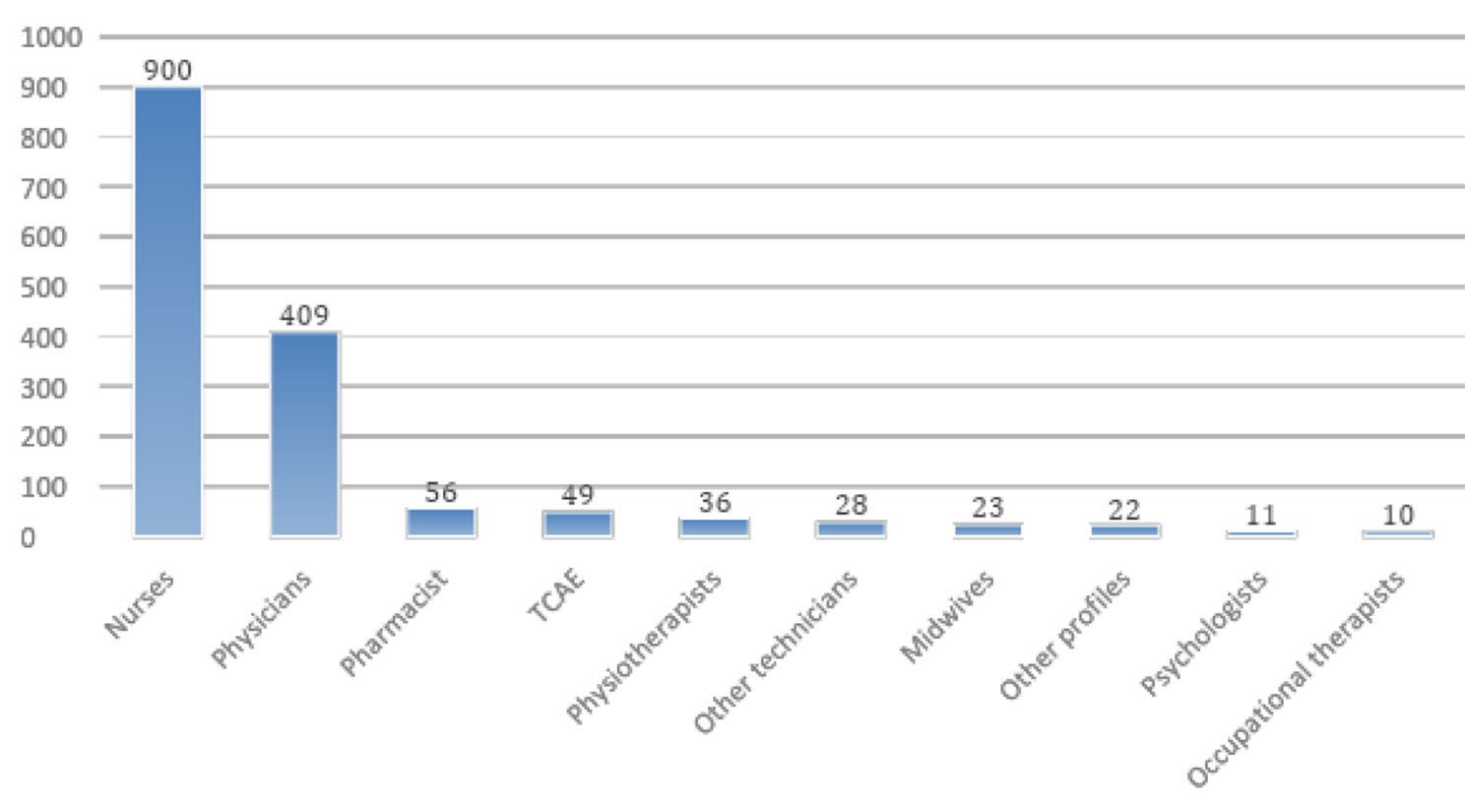
Distribution of respondent profiles

From the 1544 accepted responses, 900 were obtained from nurses, 409 from physicians, 56 from pharmacists, 36 from physiotherapists, 23 from midwives, 11 from psychologists, 49 from TCAE (auxiliary care technicians), 28 from other technicians, 10 from occupational therapists and the remaining 22 from different profiles (biologists, nutritionists, opticians, etc.). Figure 1 shows a graphical distribution of the participants’ profiles.

The most representative group was nurses, 58.29% of the total, followed by physicians 26.49%, pharmacists, and TCAE, 3.62%, and 3.17%.

According to the Spanish National Institute of Statistics [17], in 2020 (data published in 2021), there were 276,191 registered physicians and 325,018 registered nurses (45.93% physicians compared to 54.07% nurses). The necessary sample would be 384 surveys carried out by these two groups (confidence level 95%, 5% margin of error), according to the calculations mentioned above in the Methodology section. We can therefore state that the number of responses obtained is representative of both, physicians and nurses. Unlike other healthcare professionals, who participated less than doctors and nurses, namely, and therefore are under-represented in this survey.

To the question D*o you currently work in…*, nurses answered that 581 of them, (64.55%) work in hospital settings, 169 in Primary healthcare centres (18.77). 43 nurses work in universities (4.77%), 26 in out-of-hospital emergencies, 22 in both nursing homes and social-health centres. The rest work in other centres or units such as public healthcare, mental healthcare, hemotherapy, the private sector, mutual insurance companies, etc. Table 2 shows a graphical distribution of nurses and physicians depending on their work area.

**Table 2:** Distribution of nurses’ and physicians’ work areas

Work areas	Nurses	Physicians
Pharmaceutical industry	1	1
Armed Force	1	0
NGO	1	1
Public Health	2	2
Research	3	1
Schools	3	0
Hemotherapy	4	1
Mutual insurance	8	1
Management	10	5
Private sector	10	15
Mental health	16	0
Nursing homes or social health centres	22	9
Out-of-hospital emergencies	26	9
University	43	12
Primary Health Care	169	105
Hospital	581	247

To the question *Do you currently work in*… the physicians answered that 60.39% of them work in a hospital setting whereas 25.67% work in primary care centres. 15 of the participants in the survey indicated that they work in the private sector (3.67%), 12 in a university setting (2.93%), 9 in both residences and social-health centres (2.20%), and 9 in out-of-hospital emergencies (2.20%). The rest work in other centres or units such as public healthcare, hemotherapy, mutual insurance companies, etc.

We can see a similar pattern for physicians and nurses in hospitals and primary healthcare. Only 1% of those working in the management area responded.

In response to the first question in the Training received f*rom your company or organisation section, have you received any training, in recent years, related to the use of technology in the healthcare field?* 37.88% of the professionals received training in this area. Table 3 shows detailed information classified by professional profile. It can be seen from this table (Table 3) that only pharmacists and the group of other professional profiles had a higher number of people in the category “yes, I have received training”.

**Table 3:** Technology training by professional profile (Question 1)

Professional profile	Total number of professionals in each profile	I have not received training	Yes, I have received training	% of professionals who have received training about the total number of professionals
Nurses	900	589	311	20.14
Physicians	409	217	192	12.43
Pharmacists	56	23	33	2.14
Physiotherapists	36	30	6	0.38
Midwives	23	18	5	0.32
TCAE	49	39	10	0.65
Other technicians	28	19	9	0.58
Occupational therapists	10	7	3	0.19
Psychologists	11	8	3	0.19
Other profiles	22	9	13	0.84
TOTAL	**1544**	**955**	**585**	**37.88**

By isolating the physicians and nurses’ answers (1309 responses) for comparative purposes, we find that more than 60% of them have not received any training in this area (61.57%). The difference between the training received by physicians and nurses is statistically significant (p = 0.00002) Therefore, physicians receive more training in this area than nurses.

However, comparing primary care and hospital nurses and physicians, there are hardly any differences between nurses (65.68% do not receive training in primary care compared to 66.78% who do not receive it in hospitals). There is a difference between physicians working in primary healthcare and in hospitals (58.09% do not receive training in primary healthcare compared to 49.39% in hospitals). Moreover, more hospital physicians have received training than those who have not (50.60% vs. 49.39%). This data can be seen in detail in Table 4.

**Table 4:** Comparison by the workplace. Question 1

		Nurses	%	Physicians	%
Primary Health Care	No	111	65.68	61	5.09
	Yes	58	34.32	44	41.90
Hospital	No	388	66.78	122	49.39
	Yes	193	33.21	125	50.60

Answering question 2, *In your company or organisation, have you received any training in recent years related to creating healthcare content for social networks, videos, videoconferences, etc.?* Only 14.44% of the professionals received training in this area. Table 5 shows the training received in this field, categorised by professional profiles.

**Table 5:** Networking and video training by professional profile (Question 2)

Professional profile	Total number of professionals in each profile	I have not received training	Yes, I have received training	% Yes
Nurses	900	774	126	8.16
Physicians	409	348	61	3.95
Pharmacists	56	40	16	1.03
Physiotherapists	36	32	4	0.25
Midwives	23	21	2	0.13
TCAE	49	48	1	0.064
Other technicians	28	26	2	0.13
Occupational therapists	10	7	3	0.19
Psychologists	11	10	1	0.064
Other profiles	22	15	7	0.45
TOTAL	**1544**	**1321**	**223**	**14.44**

Isolating physicians and nurses’ answers (1309 responses), for comparative purposes, we find that almost 85% have not received training in creating digital content, the use of videos or social networks. The difference between the training received by physicians and nurses is not statistically significant in this case (P = 0.8689). Therefore, both groups receive little training.

Making the same comparison between physicians and nurses with question 3; From your company or organisation, have you received any training related to database searches in recent years? We find that 64.47% have not received any training in this area from their organisations for research (844 responses), with a statistically significant difference in favour of the training received by physicians compared to what was received by nurses (P = 0.0000). This data and comparison by workplace can be seen in Table 6.

**Table 6:** Comparison between physicians and nurses about Question 3

	I have not received training		Yes, I have received training	
	Nurses	Physicians	Nurses	Physicians
Primary health care	112 (66.27%)	47 (44.76%)	57 (33.73%)	58 (55.24%)
Hospital	425 (73.14%)	130 (52.63%)	156 (26.86%)	117 (4.37%)
TOTAL	634	210	266	199
	844 (64.47%)		465 (35.52%)	

Primary care physicians received more training in these subjects than their hospital counterparts (55.24% vs. 47.37%). This trend can also be found in primary care and hospital nurses (33.73% vs. 26.86%).

If we analyse the answers to question 4, including only nurses and physicians; “In your company or organisation, have you received any training related to the use of computers in recent years “, we can see that 59.82% of nurses and physicians have not received any training related to their use at the workplace. Again, the difference between the two groups is statistically significant. Consequently, physicians have received more training than nurses in computer management (P = 0.0040).

Comparing primary healthcare and hospitals the differences are less relevant than in other questions. However, it is clear that professionals working in hospitals have received somewhat more training in computer management than those in primary health care (see Table 7).

**Table 7:** Comparison between physicians and nurses to Question 4

	I have not received training		Yes, I have received training	
	Nurses	Physicians	Nurses	Physicians
Primary health care	112 (66.27%)	60 (57.14%)	57 (33.73%)	45 (42.86%)
Hospital	374 (64.37%)	127 (51.42%)	207 (35.63%)	120 (48.58%)
**TOTAL**	565	218	355	191
	783 (59.82%)		526 (40.18%)	

**Fig. 2:**
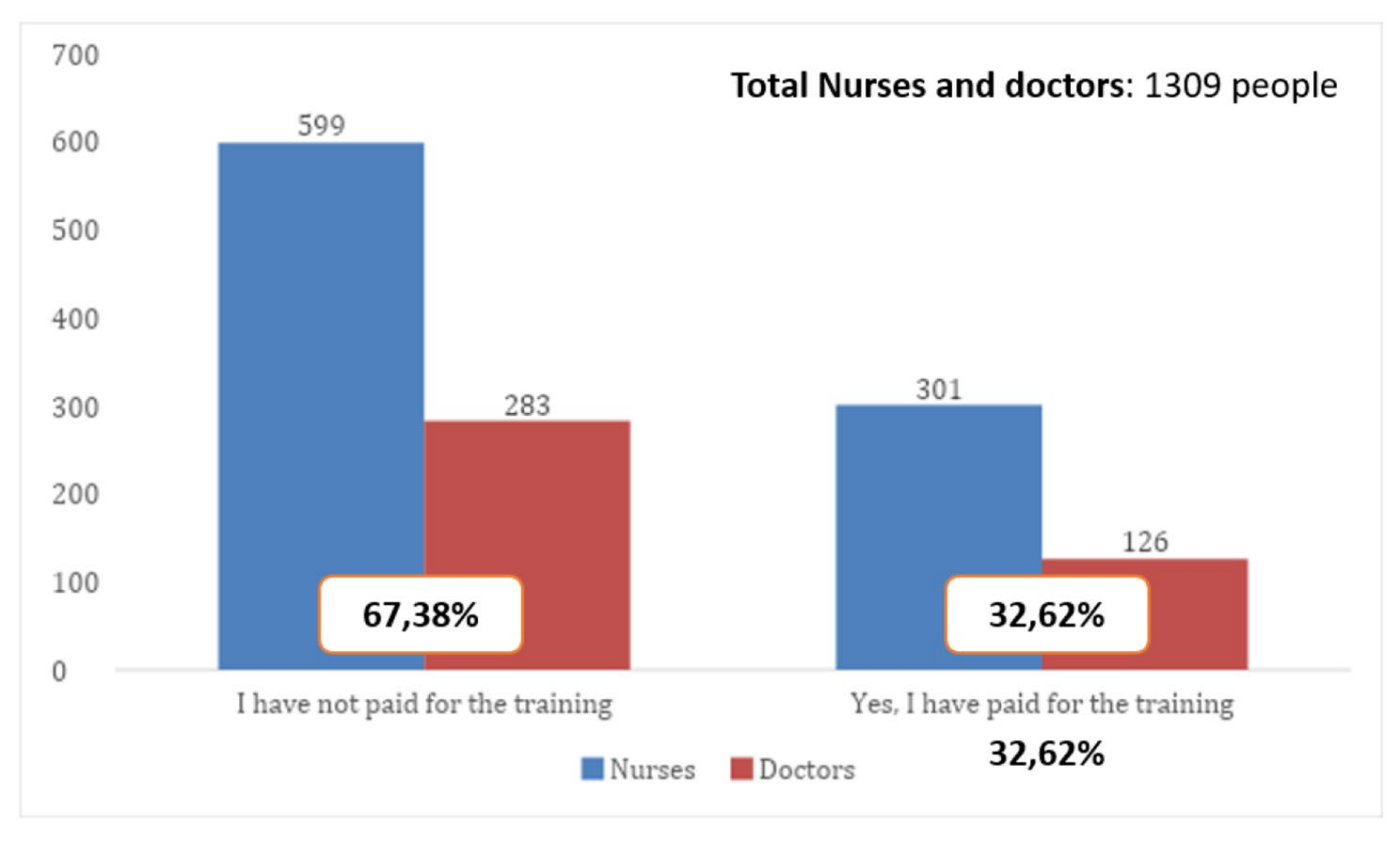
Comparison in the way training is paid for (for physicians and nurses)

Finally, in; *Have you paid for any of the courses mentioned above yourself?* (Question 5). Comparing data obtained from physicians and nurses’ responses on a whole, we found that out of the 1309 doctors and nurses who responded to the survey, 427 paid for their own training on these matters (32.62%), with no statistically significant differences between the two groups (P = 0.1905). So, it can be said that both, physicians and nurses, pay for their own training equally (see Fig. 2).

If we compare all the professional categories included in the survey, we find that psychologists, pharmacists, and occupational therapists invest the most in their own digital skills training whereas physicians and TCAEs have paid the least. However, a small number of professionals are in these categories in our sample and further studies would be necessary to confirm or debunk this.

In total, 512 healthcare professionals (33.16%) said they had financed their own training (see Table 8).

**Table 8:** Professionals who have paid for their training

Yes, I have paid for the training	Total number of individuals	% Within its category
Nurses	301	33.44
Physicians	126	30.80
TCAE	14	28.57
Midwives	8	34.78
Physiotherapists	13	36.11
Pharmacists	24	42.85
Psychologists	5	45.45
Occupational therapists	4	40
Other technicians	9	32.14
Other profiles	8	36.36
TOTAL	512	

## Discussion

This analysis aimed to depict the current situation of digital skills training offered by healthcare institutions to healthcare professionals. It was carried out with the participation of different professional profiles, most of whom were physicians and nurses. Our initial hypothesis was confirmed after analysing the data obtained. Thus, the number of healthcare professionals currently receiving training in digital skills from their organisations is low. Regarding those who receive none, physicians receive more training than other professionals like nurses. The fact that nurses receive less training in e-skills and technology management, may affect the quality of their healthcare, given that nurses need these skills to provide safe patient care [15, 18].

This trend, less training for nurses, is observed in every question, except for Question 2 (creation of digital health content), where there is no difference between physicians and nurses, only 14% have received training in this area. However, it is essential to point out that the use of videos for educational and informative purposes in health can be motivating and useful for patients, [19, 20]. It would, thus, be advisable to include this type of content in courses and training given by healthcare organisations, especially in the current pandemic where the role of professionals as mediators in filtering reliable information is more important than ever [21].

Regarding the workplace, there are no differences in training between primary care and hospital nurses, conversely, there are differences between physicians in primary care and in hospital settings. Hospital-based physicians receive the most technology and digital competence training, which could be related to the belief regarding primary care physicians not requiring technology in their clinical practice. However, there are numerous cases where using technology in the primary care setting can be implemented, such as telemonitoring patients, coordination with other clinical units, filtering relevant information, distributing quality information to the general population, etc. [22, 23].

Regarding the investigation, there is a large gap between database search training for nurses offered by healthcare institutions compared to that offered to physicians. Information seeking is a key sub-skill within digital competence that helps to locate quality information and use it responsibly. This can lead to a huge disparity in nursing research and reduce the likelihood of positive outcomes for patients and the healthcare system [24–28].

For evidence-based practice to become a reality, involving all healthcare professionals is a priority, for this reason, they must have the necessary competences [29, 30]. Similarly, to make digital health a reality and apply it in all healthcare areas, involving all healthcare professionals in training and updating programs regularly is a must [31].

Finally, it should be noted that more than 33% of the healthcare professionals surveyed paid for their own digital-related training, showing a high level of interest on their behalf, moreover, healthcare organisations are not only failing to meet the needs of their professionals but of society, especially in pandemic times, when training in digital and technological skills has become a priority [32].

## Limitations

One of this study’s limitations is the fact that participants were recruited on social networks. Having to fill in an online questionnaire could reduce the participation by people with few digital skills. The creation of this questionnaire specifically for this study should also be noted as a limitation. However, efforts have been made to achieve a sizable sample to reduce possible biases.

## Conclusions

The aim of this study was to find out whether the basic training deficiencies in digital competencies for nurses in their undergraduate training were compensated for by their employers. However, as we have seen, Spanish healthcare institutions do not train 100% of their professionals in digital competences or in the use of technology to empower patients, etc., and there is still a lot of room for improvement.

Very few healthcare professionals receive training in higher-level competences such as creating video resources or others, which hinders their applicability in clinical practice. Physicians receive most training in this area, although the number is still limited. It is important to remember that the system is multidisciplinary and requires all the agents involved to have sufficient knowledge to guarantee quality care based on the best scientific evidence.

Nurses receive less training than physicians from their healthcare centres and hospitals in research and technology and, therefore, have fewer research and digital skills, which may lead to deficits in their practice with negative effects on patients as well as fewer opportunities for professional growth.

## Recommendations for the future

As calls to action, we consider that:

1. Training for all professional profiles should be reinforced by institutions, considering that the digital competence of healthcare workers is an important asset for improving the population’s health.
2. Institutions must strengthen the research competencies of nurses through lifelong learning, monitoring and ongoing support. Nursing research can improve the quality of patient care and the professional development of nurses in their discipline.

## Data Availability

The datasets generated and/or analysed during the current study are not publicly available but are available from the corresponding author on reasonable request.
